# Lumbosacral plexopathy due to pelvic hematoma after extracorporeal membrane oxygenation: A case report

**DOI:** 10.1097/MD.0000000000025698

**Published:** 2021-04-30

**Authors:** Anson W. Wilks, Muhammad T. Al-Lozi

**Affiliations:** Department of Neurology, Washington University School of Medicine, Saint Louis, MO.

**Keywords:** electromyography/nerve conduction study, extracorporeal membrane oxygenation, lumbosacral plexopathy, pelvic hematoma

## Abstract

**Rationale:**

Peripheral nerve injury related to vascular complications associated with extracorporeal membrane oxygenation (ECMO) is perhaps underappreciated. Compared to the well-described central nervous system complications of ECMO, brachial plexopathy and lumbosacral plexopathy have rarely been reported. We report this case to heighten awareness of lumbosacral plexus injury due to pelvic hematoma formation after ECMO.

**Patient concerns:**

A 53-year-old woman developed a large pelvic hematoma with significant mass effect on intrapelvic structures after receiving lifesaving venoarterial ECMO for cardiogenic shock following a cardiac arrest. During her hospital course, she developed bilateral foot drop that was attributed to critical illness. Her lack of neurological recovery after 6 months prompted referral to neuromuscular medicine for consultation.

**Diagnosis:**

The patient was retrospectively diagnosed with bilateral lumbosacral plexopathy due to the large pelvic hematoma.

**Intervention:**

Electromyography/nerve conduction study (EMG/NCS) obtained at the time of referral to neuromuscular medicine localized her neurological deficits to the bilateral lumbosacral plexus and demonstrated no volitional motor unit action potentials in her lower leg muscles.

**Outcomes:**

The patient had minimal recovery of strength at the level of the ankles but was ambulatory with solid ankle–foot orthoses due to spared proximal lower extremity strength. Unfortunately, the absence of any volitionally activated motor unit action potentials in her lower leg muscles on EMG performed 6 months after the initial injury was a poor prognostic indicator for successful reinnervation and future neurological recovery.

**Lessons:**

Neurological deficits occurring during the course of administration of ECMO require accurate localization. Neurology consultation and/or EMG/NCS may be useful if localization is not clear. Lesions localizing to the lumbosacral plexus should prompt radiographic evaluation with computed tomography of the abdomen and pelvis. Hemostasis of a retroperitoneal hematoma may be achieved with embolization. However, if neurological deficits do not improve, surgical consultation for hematoma evacuation may be warranted.

## 1 Introduction

Neurological complications of extracorporeal membrane oxygenation (ECMO) are well known, although studies have focused on central nervous system complications such as intracranial hemorrhage, ischemic stroke, seizure, and hypoxic-ischemic encephalopathy.^[1]^ Peripheral nerve injury is perhaps underappreciated. Although retroperitoneal hematoma with resultant lumbar plexopathy or femoral neuropathy is a well-described complication of cardiac catheterization,^[2]^ peripheral nerve injury during ECMO treatment has only been described in case reports or small case series. Brachial plexopathy from hematoma formation related to axillary artery cannulation during ECMO has been described in 2 patients.^[3]^ Pelvic hematoma causing lumbosacral plexopathy during the course of ECMO has been described in a prior case report.^[4]^ We report here a case of lumbosacral plexopathy manifesting as bilateral flail foot and lower extremity sensory loss due to pelvic hematoma formation as a complication of vascular access during the administration of ECMO.

## 2 Ethics statement

Institutional review board approval was not necessary since this case report is not considered research. Written informed consent was obtained from the patient for publication.

## 3 Case report

**Figure 1:**
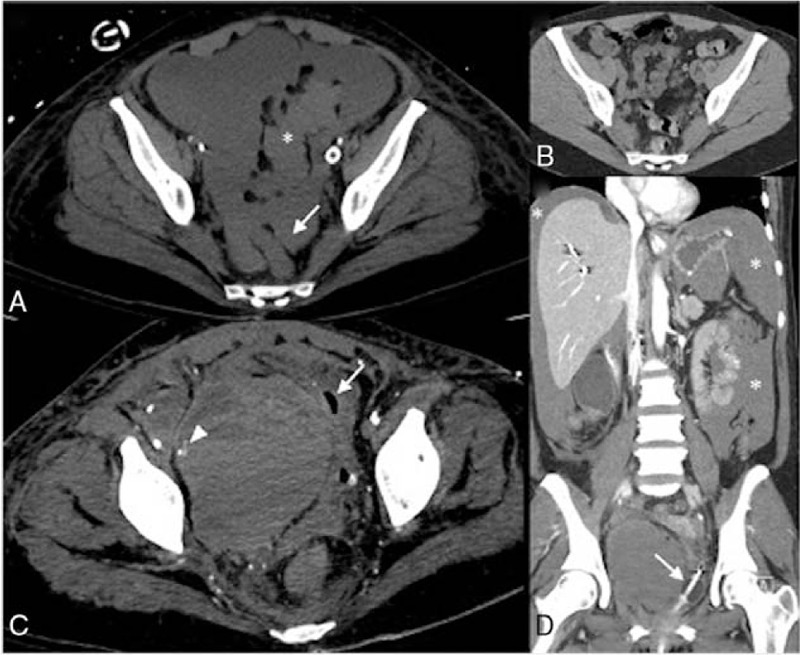
(A) Axial computed tomography (CT) at the level of the sacral promontory shows hemoperitoneum with fluid collection in the pelvis, including the retrouterine space (*arrow*), enveloping the sigmoid colon (*asterisk*). (B) Premorbid axial CT of the patient (obtained for abdominal pain) at identical level as in (A) highlights normal pelvic anatomy by means of comparison. (C) Contrast-enhanced axial CT obtained 15 d after that in (A) shows a large right-sided pelvic retroperitoneal hemorrhage with contrast extravasation (*arrowhead*), posterior compression of the rectum, and leftward displacement of the bladder with intravesical air introduced by catheterization (*arrow*). (D) Coronal reconstruction of CT from (C) shows the pelvic hematoma displacing an inflated Foley catheter (*arrow*) and highlights the large hemoperitoneum (*asterisks*) prior to its evacuation.

A 53-year-old woman presented to the hospital in cardiogenic shock after successful cardiopulmonary resuscitation for an arrest in the field. She received venoarterial ECMO, cannulated via the right femoral artery and left femoral vein. On hospital day (HD) 5, she had a significant drop in hematocrit for which computed tomography (CT) of the chest/abdomen/pelvis was obtained that identified a large intraperitoneal hemorrhage with collection in the retrouterine pouch (Fig. 1), originating from a grade IV liver injury. Embolization of a branch of the left hepatic artery achieved hemostasis. Sedation had been intermittently weaned, confirming a normal neurological exam. On HD9, ECMO was decannulated, during which the right external iliac and common femoral artery were clamped, and a complex repair of the right femoral artery was performed. On HD11, she first noticed bilateral foot drop and numbness, which were attributed to critical illness. Arm and proximal leg strength were normal. A follow-up CT chest/abdomen/pelvis performed several days later showed stability of the retrouterine hemorrhagic fluid collection. On HD20, worsening hypotension prompted a repeat CT that showed interval development of a large right-sided pelvic retroperitoneal hematoma associated with contrast extravasation and mass effect on the bladder (Fig. 1) and small bowel wall thickening concerning for ischemic bowel. An exploratory laparotomy was performed during which small bowel resection and hemoperitoneum evacuation were performed. Interventional radiology addressed the intercurrent pelvic retroperitoneal hematoma by stenting an identified right external iliac artery pseudoaneurysm and embolizing the source of the hemorrhage, an irregular distal branch of the right internal iliac artery. Since the stability of the pelvic retroperitoneal hematoma was confirmed with repeat imaging, surgical evacuation of the hematoma was not pursued. On HD37, she developed hematuria, for which cystography was performed that showed bladder rupture due to mass effect from the pelvic hematoma (Fig. 1). This was managed conservatively by urology. After a 10-week hospitalization, she was discharged with significant improvement in cardiac function.

**Figure 2:**
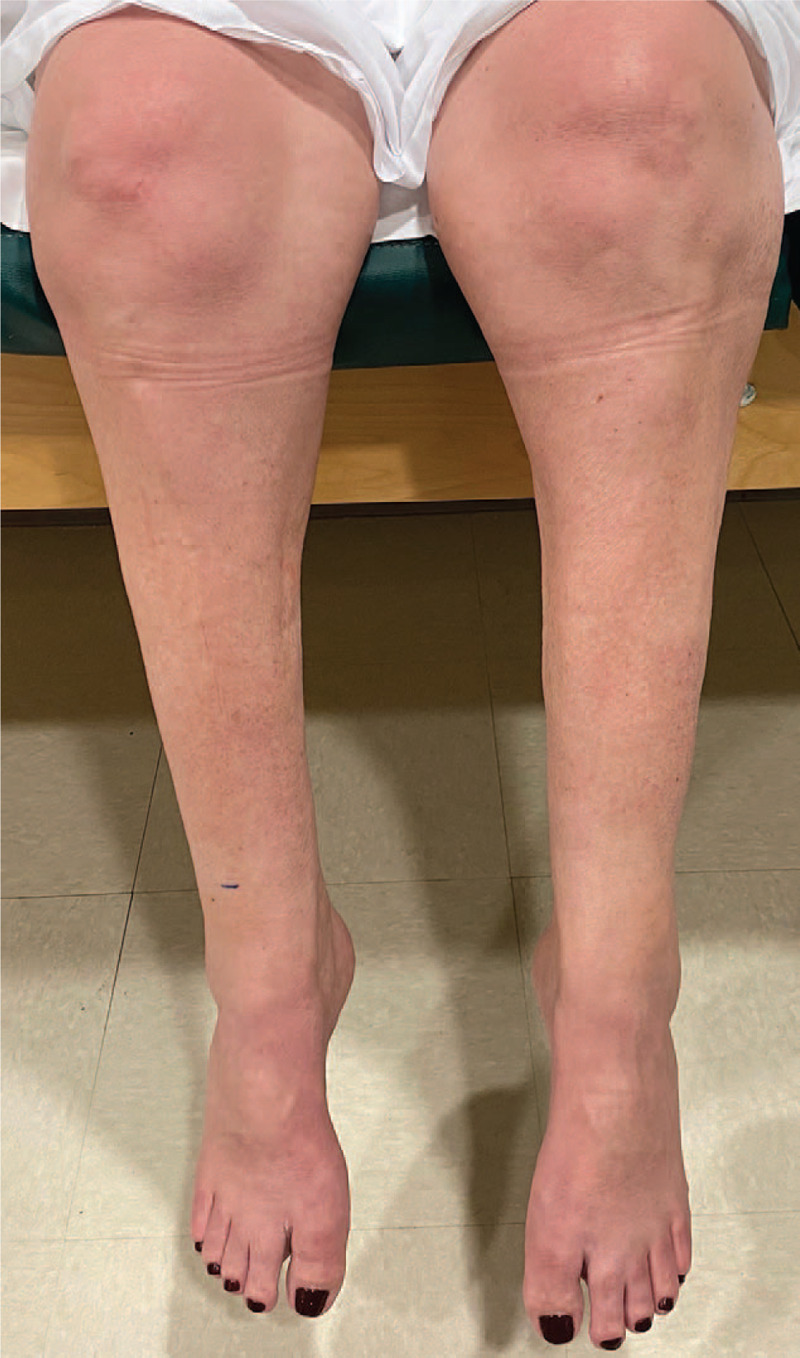
Bilateral flail foot and severe atrophy of anterior and posterior leg compartments.

The lack of improvement in lower extremity strength after 6 months prompted neuromuscular medicine consultation. The patient had no volitional movement at the level of the ankles, due to which she required solid ankle–foot orthoses to ambulate. She also reported numbness and dysesthesia below the knee bilaterally, saddle anesthesia, and decreased awareness of bowel and bladder distension. Examination confirmed bilateral paralysis and pan-modal sensory loss below the knees, leg atrophy (Fig. 2), weakness (Medical Research Council scale: 4/5) of hip adduction and abduction and knee flexion, and absence of Achilles reflexes. Hip flexion, knee extension, and upper extremity strength were normal. A nerve conduction study (NCS) showed absent bilateral sural sensory nerve action potentials (SNAPs) and tibial H-reflexes (including direct muscle responses). Motor NCSs performed on the right showed no tibial or peroneal (including from the tibialis anterior) responses. Electromyography (EMG) performed on the right showed abundant fibrillations without any volitional motor unit action potentials (MUAPs) in the tibialis anterior and medial gastrocnemius; fibrillations in the tensor fasciae latae and reduced recruitment of high-amplitude, long-duration MUAPs in both the tensor fasciae latae and gluteus maximus; normal recruitment of mildly prolonged-duration, polyphasic MUAPs in the adductor longus; and normal lumbar paraspinal and iliopsoas muscles. The absence of volitional MUAPs implied discontinuity of nerve to the lower leg muscles, which after 6 months post-injury is a poor prognostic sign for spontaneous reinnervation and neurologic recovery.^[5]^ The above electrodiagnostic findings were consistent with bilateral lumbosacral plexopathy, attributed retrospectively to mass effect from pelvic hematoma due to vascular injury occurring in the course of lifesaving ECMO.

## 4 Discussion

Since its first use in the 1960s as respiratory support for critically ill neonates, ECMO has been utilized in both the pediatric and adult patient population and for both respiratory and cardiac indications. Indeed, in recent years there has been a significant increase in utilization of ECMO due to studies that have shown a survival benefit,^[6,7]^ and it is often recommended at centers with experience in providing ECMO for patients with acute respiratory or cardiac failure refractory to conventional management. Venoarterial ECMO, as in our case, is typically used for cardiac failure while venovenous ECMO is used for respiratory failure. As is to be expected with any invasive procedure, complication rates are high. Neurological complication rates vary depending on the study and definition utilized and range from 4% to 37%.^[8]^ Long-term neurological sequelae are not as well characterized in the literature. One study, however, found that severe neurological sequelae occurred in up to 50% of adult ECMO-treated patients.^[1]^ Most of these neurological sequelae involve the central nervous system.

Retroperitoneal hematoma causing lumbar plexopathy or femoral neuropathy has been well described, particularly secondary to therapeutic anticoagulation^[9,10]^ or during cardiac catheterization.^[2]^ It follows that hematoma formation would be an expected complication of ECMO. Indeed, hemorrhage is one of the most common complications encountered during ECMO.^[11]^ Nonetheless, while retroperitoneal hematoma is a known complication, peripheral nerve injury related to this during ECMO may be underreported. We, in our literature review, only found 1 case report describing lumbosacral plexopathy due to hematoma formation associated with ECMO.^[4]^

Pelvic hematoma, unrelated to ECMO, has been described to occur secondary to pelvic trauma^[12]^ or rupture of an iliac artery pseudoaneurysm.^[13,14]^ It may cause lower lumbosacral plexopathy (i.e., lumbosacral trunk and sacral plexus involvement), which manifests as dropped foot or flail foot, as in this case. This neurological syndrome is potentially a less recognizable complication of retroperitoneal hemorrhage compared to that of the more common iliopsoas retroperitoneal hematoma (i.e., weakness of hip flexion and knee extension and depressed patellar reflex). This atypical clinical picture of foot drop may have added to diagnostic confusion in our case.

Any neurological deficit occurring during the course of ECMO requires immediate evaluation. Localization, with the assistance of EMG/NCS in appropriate cases, will guide diagnostic studies, including imaging, such as CT of the abdomen and pelvis. Hemostasis of an identified retroperitoneal hematoma is paramount, as in this case. However, neurological injury related to mass effect from the hematoma should be considered as well. If neurological deficits fail to improve, surgical consultation may be warranted as early relief of mass effect from the hematoma may reverse or alleviate neurological injury in these extraordinary cases.^[15]^

## Author contributions

**Conceptualization:** Anson W. Wilks, Muhammad T. Al-Lozi.

**Data curation:** Anson W. Wilks, Muhammad T. Al-Lozi.

**Formal analysis:** Anson W. Wilks, Muhammad T. Al-Lozi.

**Supervision:** Muhammad T. Al-Lozi.

**Writing – original draft:** Anson W. Wilks.

**Writing – review & editing:** Muhammad T. Al-Lozi.
